# Supplementary material for: Patterns of regulatory behavior in the still-face paradigm at 3 months: A comparison of Brazilian and Portuguese infants
Source: PLoS One. 2021 Jun 11;16(6):e0252562. doi: 10.1371/journal.pone.0252562 (PMC8195415; doi:10.1371/journal.pone.0252562)
Supplement: Attachment — Submitted filename: response to reviewers plos one final.docx [file pone.0252562.s001.docx]

April 22, 2021

**PONE-D-20-40216
Patterns of Regulatory Behavior in the Still-Face Paradigm at 3 months: A Comparison of Brazilian and Portuguese Infants
PLOS ONE**

**Dear Editor of PLOS ONE - Professor Julie Jeannette Gros-Louis**

The authors would like to thank the reviewers for their very helpful critiques and suggestions, and for allowing us the opportunity to revise our manuscript. We have carefully attended to, and have done our best to implement or address, each of your suggestions and comments. We have revised the manuscript in detail and edited according to each suggestion.

Starting with the PLOS ONE editorial indications:

1. We ensured that the manuscript was formatted according PLOS ONE's style requirements.

2. We included the title page within the main document listing all authors and affiliations.

3. We corrected the grant information provided in the ‘Funding Information’ and ‘Financial Disclosure’. Now, both sections match.

4. We amended the manuscript submission data to include author Rita Almeida and included this author in the authorship list.

6. We reviewed all overlaps with our previous publications using the software URKUND, and included the respective citations or reworded the text.

7. We included our data set for open access in URL https://osf.io/ymqcj.

**First reviewer**

**Overall Comments.** We are grateful for the first reviewer for his/her significant suggestion and critiques. We agree with the suggestion regarding the introduction. Therefore, we reviewed the Introduction to explain why differences in infants’ reaction to the Still Face Task might emerge in the chosen countries and added study hypotheses. We hope the revised version of manuscript addresses better the cultural, economic, and social distinctions between both countries (in the introduction, present study and discussion) and their associations of each with infant regulatory patterns and maternal sensitivity. For that purpose, we also added new results for maternal sensitivity in both samples in Table 4 and we add new section of results “Association between maternal behavior and infant regulatory patterns”. We are also grateful for the suggestion about providing greater specification and information about the three infant patterns (Social-Positive, Distressed-Inconsolable, Self-Comfort) in the Methods section in table 2.

Also according to the reviewer suggestion:

**Introduction**
1. We clarified the information regarding the behavior of infants with Distressed-Inconsolable pattern across FFSF in page 4 last two sentences and page 5 first sentence.

2. In the Chiodelli et al. (2020) study, infants’ had difficulty in recovering from negative affect due to maternal intrusiveness during the reunion episode. We detailed this result on page 6 - line 16.

3. Greater detail about specific differences and similarities between Brazil and Portugal are now provided in section Present Study - pages 6-8.

**Method**
4. We provided more detail about the specific behaviors associated with the three infant regulatory patterns in each episode of the FFSF, and added a new table summarizing this information, on page 13 and 14. We agree that adding this information, the text is clearer and more exact.

5. We provided more detailed information about the coding system in pages , including the dimensions coded, quality of behaviors, and the six sub-categories, on page 13 (line 5-10; 10-16) and 14 (line 6-10) and in Table 2.

**Results**
6. We clarified what by “other national samples” we meant Portuguese Samples. We corrected this error in page 16 line 15 and we included a reference.

7. Table 3. We agree that the percentages of infants in each regulatory pattern are the most meaningful data in the table because the number of infants differed in the two samples. We accepted the suggestion to place the percentages first and put the number of infants in parentheses. We agree that this approach is clearer and easier to read in the table.

8 and 9. We reframed the present study section to include the study hypotheses (page 8 line 5 and line 13).

10. The reviewer stated: *“Although the authors allude to the cultural differences in infants’ responsive patterns may be due to differences in SES and infant health at birth, I suggest these possibilities be made more explicit. Portuguese mothers had higher SES than Brazilian mothers and their infants showed predominantly the Social-Positive pattern; Brazilian mothers with higher SES had infants who more likely showed the Social-Positive pattern. Likewise, Brazilian infants had lower Apgar scores than Portuguese infants and showed predominantly the Distress-Inconsolable pattern; Portuguese infants with lower Apgar scores more likely showed the Distress-Inconsolable pattern. Thus might SES and infant health at birth be possible explanation for the results?”* We agree with this statement and have reframed substantially this section of discussion, to address the specific questions formulated.

11. We included additional evidence for differences in parenting between Portuguese and Brazilian samples on table 4, results section page 16 (starting in line 20) and page 18. Also these new results were discussed and introduced in the abstract.

12. We changed “The authors suggest, may reflect…”. for “The authors suggest that the differences reflect …” in page 6 line 5.

13. We changed “one focuses” to “one focused, in page 12 line – 23 and 24.

**Second reviewer**

**Overall suggestions**. We are grateful for the reviewer for his/her appreciation of our manuscript and significant suggestion, particularly to add new results regarding maternal behavior in both samples. New results are now provided on table 4, results section page 16 (starting in line 20) and page 18. To explain the methodology used, we add a new section in methods named free play interactions. Also these new results were discussed and introduced in the abstract. . We believe that this new information helped us to better discuss the differences found between both samples and overall improved the manuscript.

1. More detail information about the coding system (the dimensions coded, quality of behaviors, the six sub-categories) is presented in page 13 (line5-10; 16-19) and 14 (line 6-10) and in Table 2.

2. We provided more detailed information about intercoder reliability for both the coding of maternal behavior during free play and the coding of infant regulatory patterns. We clarified that coding was carried out independently in each sample, by a team of trained reliable coders who were masked to the study’s hypotheses and background information. We also provided information about the number of coders in each team and what percentage of videos were coded for reliability by sample (see page 12 line 5-13 and page 14 line 10-14). We agree that adding this information makes the description of coding procedures more detailed and exact.

**Final comments**. We also revised the manuscript for typos and errors. Since we included maternal behavior in the analyses we excluded three Brazilian mothers that had less than 18 years old and analyses were updated.
